# Volatiles of fungal cultivars act as cues for host-selection in the fungus-farming ambrosia beetle *Xylosandrus germanus*

**DOI:** 10.3389/fmicb.2023.1151078

**Published:** 2023-04-14

**Authors:** Antonio Gugliuzzo, Jürgen Kreuzwieser, Christopher M. Ranger, Giovanna Tropea Garzia, Antonio Biondi, Peter H. W. Biedermann

**Affiliations:** ^1^Department of Agriculture, Food and Environment, University of Catania, Catania, Italy; ^2^Ecosystem Physiology, University of Freiburg, Freiburg, Germany; ^3^Horticultural Insects Research Laboratory, USDA-Agricultural Research Service, Wooster, OH, United States; ^4^Chair for Forest Entomology and Protection, University of Freiburg, Stegen, Germany

**Keywords:** *Ambrosiella grosmanniae*, fungal volatiles, mutualism, MVOCs, Symbiosis, aggregation pheromone, Xyleborini

## Abstract

Many wood-boring insects use aggregation pheromones during mass colonization of host trees. Bark beetles (Curculionidae: Scolytinae) are a model system, but much less is known about the role of semiochemicals during host selection by ambrosia beetles. As an ecological clade within the bark beetles, ambrosia beetles are obligately dependent on fungal mutualists for their sole source of nutrition. Mass colonization of trees growing in horticultural settings by exotic ambrosia beetles can occur, but aggregation cues have remained enigmatic. To elucidate this mechanism, we first characterized the fungal associates of the exotic, mass-aggregating ambrosia beetle *Xylosandrus germanus* in Southern Germany. Still-air olfactometer bioassays documented the attraction of *X. germanus* to its primary nutritional mutualist *Ambrosiella grosmanniae* and to a lesser extent another common fungal isolate (*Acremonium* sp.). During two-choice bioassays, *X. germanus* was preferentially attracted to branch sections (i.e., bolts) that were either pre-colonized by conspecifics or pre-inoculated with *A. grosmanniae*. Subsequent analyses identified microbial volatile organic compounds (MVOCs) that could potentially function as aggregation pheromones for *X. germanus*. To our knowledge, this is the first evidence for fungal volatiles as attractive cues during host selection by *X. germanus*. Adaptive benefits of responding to fungal cues associated with an infestation of conspecifics could be a function of locating a suitable substrate for cultivating fungal symbionts and/or increasing the likelihood of mating opportunities with the flightless males. However, this requires solutions for evolutionary conflict arising due to potential mixing of vertically transmitted and horizontally acquired symbiont strains, which are discussed.

## 1. Introduction

Several crucial biological processes in insects, such as partner-choice, foraging, dispersal and many others, are mediated by olfaction (Fleischer et al., 2018; Xu and Turlings, 2018; Schmidt and Benton, 2020). Semiochemicals involved in these processes comprise pheromones for intraspecific communication and allelochemicals for interspecific communication (Norin, 2007; Davis et al., 2013; Cale et al., 2016; Guo and Wang, 2019; Bueno et al., 2020; Crowley-Gall et al., 2021; Weisskopf et al., 2021). Interestingly, an increasing number of studies demonstrate that some insect pheromones, in some cases, are not derived from insects but are instead produced by microbial symbionts (Carthey et al., 2018; Engl and Kaltenpoth, 2018; Ren et al., 2021).

Behavioral responses to microbial volatile organic compounds (MVOCs) are widespread in many major insect groups (Davis et al., 2013). Microbially produced volatiles that facilitate the detection of food and/or breeding substrate can be classified as allelochemicals, i.e., kairomones, allomones, and synomones (Wertheim et al., 2005; Davis et al., 2013; Engl and Kaltenpoth, 2018). Alternatively, MVOCs that facilitate the location of conspecifics for mating and other beneficial aggregations can be regarded as aggregation pheromones, despite their interspecific communication properties (Wertheim et al., 2005; Davis et al., 2013; Engl and Kaltenpoth, 2018). A growing body of research demonstrates that insects use MVOCs as pheromones (Davis et al., 2013; Engl and Kaltenpoth, 2018). For instance, aggregation pheromones that drive mass aggregations to host trees by tree-killing bark beetles (Curculionidae: Scolytinae) are produced, in part, by the beetles’ bacterial and fungal symbionts (Xu et al., 2015; Zhao et al., 2015; Netherer et al., 2021). These microbial symbionts also help to localize nutrients within host tissues and are considered essential for the colonization of living trees (Engl and Kaltenpoth, 2018; Netherer et al., 2021).

Ambrosia beetles in the tribe Xyleborini (Scolytinae), which are an ecological clade within the bark beetles, tunnel into host trees for the purposes of cultivating their nutritional fungal mutualists (Hulcr and Stelinski, 2017; Diehl et al., 2022). The role(s) that MVOCs play within the fungus-farming ambrosia beetles is emerging, but could function as short- or long-range cues to indicate the suitability of host substrates for brood production (Hulcr et al., 2011; Kuhns et al., 2014; Egonyu and Torto, 2018; Kandasamy et al., 2019; Biedermann and Vega, 2020; Ranger et al., 2021a; Nones et al., 2022). Some xyleborine species in the genera *Xylosandrus* and *Euwallacea* (Scolytinae) exhibit mass aggregations on recently dead or dying trees (Hulcr and Stelinski, 2017). The emission of ethanol from physiologically-stressed trees acts as an important long- and short-range cue for many xyleborine ambrosia beetles, but MVOCs could also play a role during the host-selection process (Gugliuzzo et al., 2021; Ranger et al., 2021a,b). For instance, the redbay ambrosia beetle, *Xyleborus glabratus*, is more attracted to volatile emissions from swamp bay *Persea palustris* (Raf.) Sarg. infected with the beetles’ fungal symbiont *Harringtonia lauricola* (T.C. Harr. et al.) Z.W. de Beer and M. Procter compared to healthy plants (Martini et al., 2017; de Beer et al., 2022). Characterizing attractants and repellents is a necessary component for developing “push-pull” management tactics against *Xylosandrus* spp. and *Euwallacea* spp. ambrosia beetles (Hulcr and Stelinski, 2017; Gugliuzzo et al., 2021; Ranger et al., 2021b).

Most studied ambrosia beetles vertically transmit mutualistic fungi (mainly in the genera *Ambrosiella*, *Harringtonia*, *Dryadomyces*, *Raffaelea*, and *Fusarium* (Ascomycota)) in dedicated fungus-spore-carrying structures (mycangia or mycetangia) (Vega and Biedermann, 2020; de Beer et al., 2022; Mayers et al., 2022). A nutritional role as unique and specific source of nutrition for the developing progeny, is attributed to these species-specific cultivars (Norris and Baker, 1968; Lehenberger et al., 2021). Indeed, adult females inoculate spores of the fungal cultivars on the walls of tunnels dug into the host plant xylem and oviposition occurs only after the beetle foundress feeds on the established fungal mycelium (Biedermann and Vega, 2020). On the other hand, several other microorganisms have commonly been found in association with ambrosia beetles, including commensals, potential antagonists, and plant pathogens (Rassati et al., 2019; Grubbs et al., 2020; Gugliuzzo et al., 2020, 2022). However, apart from vertical transmission of cultivars within mycetangia and fungal screening through tree-host choice (e.g., Ranger et al., 2018), other mechanisms involved in the preservation of specific insect-fungal relationships like behavioral partner choice remain unstudied in ambrosia beetles. The profile of MVOCs emitted from a fungal mutualist could help to maintain specific beetle-symbiont partner choices.

*Xylosandrus germanus* (Blandford) (Coleoptera: Curculionidae: Scolytinae) is an ambrosia beetle native to subtropical Asia and currently established in parts of Europe and North America (Galko et al., 2019; Dzurenko et al., 2021). This ambrosia beetle is obligately associated with its nutritional fungal mutualist, *Ambrosiella grosmanniae* C. Mayers, McNew and T.C. Harr., on which the larvae and adults must feed to properly develop and reproduce (Microascales: Ceratocystidaceae) (Mayers et al., 2015; van de Peppel et al., 2018). Other microbial associates have been described from *X. germanus* with unknown roles (Agnello et al., 2017; Ito and Kajimura, 2017; Tuncer et al., 2017; Contarini et al., 2020). A recent study demonstrated, by means of still-air walking bioassays, an arrestment response by *X. germanus* individuals to volatiles of *A. grosmanniae* (Ranger et al., 2021a). Similarly, two other species in the genus *Xylosandrus*, namely, *X. crassiusculus* and *X. compactus*, showed attraction toward volatiles emitted by their fungal symbionts during olfactometer assays (Hulcr et al., 2011; Egonyu and Torto, 2018). To the best of our knowledge, the aforementioned studies are the only three focusing on interaction between volatiles of fungal symbionts (growing *in vitro*) and a *Xylosandrus* species.

The aim of the current study was to elucidate the influence of MVOCs produced by beetle associated fungi during tree-host colonization on the orientation and host selection behavior of *X. germanus*. To this end, we first isolated and characterized beetle associated microorganisms from wild-caught dispersing *X. germanus*. The capacity of *X. germanus* to differentiate the MVOCs of three fungal isolates was then tested in dual choice bioassays. Next, dual choice bioassays were used to measure the preference of *X. germanus* for host tissues pre-infested by a conspecific foundress or pre-infected by the fungal symbiont *A. grosmanniae* as compared to control tissues. Finally, VOCs were collected and identified from pre-infested and pre-infected host tissues.

## 2. Materials and methods

### 2.1. Study system

Attacks of *X. germanus* can occur in various ecosystems, including forests, plant nurseries, orchards, and urban areas (Ranger et al., 2016; Gugliuzzo et al., 2021). Over 200 plant species are included in the broad host range of *X. germanus* (Galko et al., 2019). Thin-barked deciduous species in the early stages of physiological stress are preferred within ornamental nurseries by dispersing *X. germanus* (Ranger et al., 2021b). As most ambrosia beetles belonging to the tribe Xyleborini, *X. germanus* males are flightless, smaller than females and do not possess mycetangia (Rabaglia et al., 2006). Females reproduce through haplodiploidy and mating between siblings inside maternal galleries is predominant. Production of a long-range sex or aggregation pheromone by *X. germanus* is not expected due to haplodiploid reproduction and flightless males. Instead, dispersing *X. germanus* females rely on the emission of stress-induced volatiles, particularly ethanol, to locate suitable hosts (Ranger et al., 2021b). Ethanol represents the most active long-range attractant tested to date for *X. germanus*, while other potential semiochemicals showed inconsistent activity when tested alone or in combination with ethanol (Ranger et al., 2021b).

### 2.2. Beetle collection and isolation of associated fungi

Dispersing *X. germanus* females were collected using ethanol-baited traps (Steininger et al., 2015) deployed in Stegen-Wittental, Freiburg, Germany (47°59′27”N 7°56′30″E) between April and June 2021. Bottle-traps (Supplementary Figure S1) were exposed within a natural forest with a mixed species composition including European beech (*Fagus sylvatica* L.), Norway spruce (*Picea abies*), pine (*Pinus* spp.), oak (*Quercus* spp.), and fir (*Abies* spp.). A total of 24 ethanol-baited traps were deployed at about 100 m distance from each other. Traps were affixed ~80 cm above the ground to the trunk of beech trees (Supplementary Figure S1). Female *X. germanus* were collected from the traps at 3–5 day intervals after which they were immediately placed in individual 1.5 mL Eppendorf tubes with a piece of moistened sterile tissue. Specimens were then transported to our laboratory at the University of Freiburg for isolation of associated fungi. Ethanol lures were replenished during each trap inspection.

The assessment of the beetle associated fungal community was conducted using 21 wild-caught female *X. germanus*. Fungal symbionts were isolated by first grinding individual females in a sterile PBS buffer solution (137 mM NaCl; 2.7 mM KCl; 10 mM Na_2_HPO_4_; 1.8 mM KH_2_PO_4_; 0.1% Tween-20, pH 7.4). The obtained mixture was then diluted (1:10, 1:100, 1:1000) and 200 μL of each dilution were spread on YEMA (yeast extract malt agar) plates. Three plates were setup for each of the three dilutions and for each specimen. Plates were incubated in the dark at 25°C until fungal colonies (CFUs) appeared. After 6 days, the dilution 1:100 was chosen as the best for counting CFUs. For each plate (1:100 dilution, *n* = 63), CFUs showing different morphology were counted and the relative proportions calculated (averaged individually among the 21 tested individuals). Moreover, also CFUs of both yeasts and bacteria were counted and their average among replicates calculated.

### 2.3. DNA extraction and characterization of fungal isolates

Pure cultures of all isolated fungal morphotypes were grown for 5 days on sterile cellophane in YEMA plates in order to more easily harvest fungal mycelium for DNA extraction. For each fungal isolate, a constant amount of actively growing mycelium (about 2 mm^2^) was placed in a 1.5 mL sterile Eppendorf tube and tDNA was extracted using the Animal and Fungi DNA Preparation Kit by Jena Bioscience, Germany. To identify the different fungi, the ITS region was amplified using the primers ITS1-F (TCCGTAGGTGAACCTGCGG) and ITS4-R (TCCTCCGCTTATTGATATGC) (White et al., 1990). For all PCR reactions we used the following master mix for 50 μL: 25 μL 2x phusion high-fidelity PCR master mix with GC buffer (Thermo Scientific™, Germany), 2.5 μL forward primer (10 μM, Eurofins Genomics, Germany), 2.5 μL reverse primer (10 μM, Eurofins Genomics, Germany), 18 μL ddH_2_O, 2 μL template (usually 1:10 diluted). The following PCR conditions were applied: 95°C for 3 min, followed by 38 cycles at 95°C for 1 min, 58°C for 1 min and 72°C for 2 min, ending with 72°C for 10 min including a storage temperature of 10°C. The purification of the obtained PCR products was performed by means of a PCR Purification Kit by Jena Bioscience, Germany after performing gel electrophoresis. Sequencing was carried out by Eurofins (Eurofins Genomics, Germany). The identification of the fungal species was done using BLASTn at NCBI (Altschul et al., 1990). All obtained sequences were uploaded to the NCBI GenBank database.

### 2.4. Laboratory rearing of *Xylosandrus germanus*

Wild-caught *X. germanus* collected in 2020 within the previously described habitat were reared under laboratory conditions for two consecutive generations prior to being used in our experiments. Laboratory rearing was conducted using a beech sawdust-based artificial diet as described in Biedermann et al. (2009) and based on Peer and Taborsky (2004). Briefly, the artificial diet consists of 100 g beech sawdust, 0.63 g Wesson’s salt mixture, 2.5 g yeast, 2.5 g casein, 5 g starch, 2.5 g sucrose, 15 g agar, 5 mL wheat germ oil, and 4 mL of 95% ethanol. After autoclaving at 121°C for 20 min, the media inside the tubes (18 by 150-mm culture tubes) is compacted using a sterile spatula and the tubes are allowed to dry for 24 h within a laminar flow hood. Female *X. germanus* are surface sterilized by a brief dip (~ 5 s) in 70% ethanol and deionized water to reduce the presence of microbial contaminants occurring on the beetle body surface. Next, beetles are individually released into the rearing tubes, which are loosely capped and positioned horizontally within an incubating room (0.24 h L:D, 25 ± 1°C, 60 ± 10% RH). Consecutive rearing is done by collecting emerging adult females from the substrate surface in rearing tubes (Biedermann et al., 2009).

Freshly eclosed beetles collected from the rearing tubes were immediately transferred to Petri dishes containing sterile, moistened filter paper and held at room temperature for 12 h. Beetles were then transferred to a new Petri dish containing sterile, moistened filter paper for another 12 h to further facilitate the loss of microbial contaminants attached to the beetle’s exoskeleton. These beetles were then surface sterilized and used in behavioral bioassays.

### 2.5. Behavioral bioassays

#### 2.5.1. Fungal volatile dual choice bioassays

Behavioral bioassays were carried out within experimental arenas to assess the behavioral response of *X. germanus* to VOCs of predominant fungi isolated from dispersing adults. These fungi consisted of the primary nutritional symbiont *A. grosmanniae* and the second most occurring beetle associated fungus *Acremonium* sp. A *Cladosporium* sp. was used as a negative control in bioassays to represent a non-symbiont contaminant. Since the biological function of the bacterial and yeast community isolated from *X. germanus* is not known to date, and considering the known obligate nutritional dependency on fungal cultivars, we conducted behavioral bioassays only with beetle associated fungi. Bioassays were conducted using a still-air olfactometer partially adapted from Kandasamy et al. (2019). The experimental arena consisted of a 9 cm diameter circular Petri dish with three Eppendorf tubes (1.5 mL, 10.7 × 38.9 mm, diameter × height) inserted at the dish base and with the tube lids reaching the lid of the dish. Tubes were equidistant from each other and placed 0.5 cm from the outer edge of the Petri dish. Two holes (diameter ~ 3 mm) were drilled at the opposite sides of two of the three tubes to allow the beetles to enter the tube during the bioassay. The other tube had the function to keep the plate in a horizontal position during the test and was left empty and without holes. To allow ventilation, eight and four small holes (diameter ~ 1 mm) were additionally provided on the walls and on the lid (see Supplementary Figure S2) respectively.

Fungal isolates were grown individually on PDA for 6 days before being used for the bioassays. A section (0.5 × 0.5 cm, l × w) of PDA with actively growing fungi, or without mycelia as a control, was placed within individual Eppendorf tubes (Supplementary Figure S2). The following two-choice comparisons were tested: (1) *A. grosmanniae* vs. control, (2) *Acremonium* sp. vs. control; (3) *Cladosporium* sp. vs. control, and (4) *A. grosmanniae* vs. *Acremonium* sp. One *X. germanus* was released in the center of each dish and the choice among the two odor sources was monitored at 30 min, 1 h, and 3 h after exposure. There were 40 replicates (i.e., 40 different beetles) tested for each combination of odor sources. Bioassays were conducted within an isolated room maintained at 0:24 h L:D, 23 ± 2°C, and 60 ± 10% RH during the experiment. A choice was considered valid when the beetle fully entered into a particular Eppendorf tube. After 3 h, beetles that were not found inside one of the two tubes were recorded as having made no choice and excluded from data analysis.

#### 2.5.2. Beetle preference for pre-infested or pre-infected wood

The effect of wood pre-infestation by conspecifics or wood pre-infection by the primary fungal mutualist *A. grosmanniae* on the preference behavior of *X. germanus* was evaluated using a two-choice, still-air arena (Supplementary Figure S3). For bioassay purposes, healthy beech branches (10–14 mm diam.) were cut into ~8 cm long sections, sealed at both ends with Parafilm®, and soaked in 10% ethanol for 2 h to promote burrowing by *X. germanus*. Soaked bolts were allowed to air dry for 30 min and then used immediately in bioassays.

To test the effect of pre-infestation on *X. germanus* preference, two bolts were first placed at opposite sides within the quadratic bioassay arena and one beetle was released for each replicate. The foundress beetle was allowed to burrow into one of the bolts for 6 days to cultivate its fungus and lay eggs (Supplementary Figure S4A). A second beetle was then released into the same arena and its preference to initiate burrowing in the pre-infested vs. non-infested control bolt was monitored after 1 h, 3 h, 24 h, and 48 h.

The effect of wood pre-infection on *X. germanus* choice was tested by first inoculating bolts with the primary beetle mutualist *A. grosmanniae*. A single, artificial hole (~5 mm deep and ~ 2 mm wide) was bored into the center of all bolts using a rotating cutting tool (Dremel^®^, Robert Bosch Tool Corporation, Germany). A mycelial plug (~2 mm wide) of 6 days old, actively growing *A. grosmanniae* was then placed into the drilled hole. Control bolts received a drilled hole and a non-inoculated piece of PDA. Bolts inoculated with *A. grosmanniae* vs. a non-inoculated control were then placed at opposite sides of the quadratic arenas (Supplementary Figure S3). After 6 days of incubating, when the *A. grosmanniae* mycelia was confirmed to have grown around the drilled hole of the artificially-inoculated bolt (Supplementary Figure S4B), one *X. germanus* was released inside the arena and burrowing preference between the pre-infected vs. non-infected bolt was monitored at 1 h, 3 h, 24 h, and 48 h.

A total of 60 females and odor sources were tested for each choice combination consisting of pre-infested vs. non-infested bolt and pre-infected vs. non-infected bolt. Arenas were held in an isolated room at 0:24 h L:D, 25 ± 1°C, and 70 ± 5% RH. The preference of each tested *X. germanus* was considered valid when the beetle initiated boring into one of the two bolts. After 48 h, beetles that did not initiate boring into one of the two bolts were excluded from data analysis.

### 2.6. Collection and identification of microbial VOCs

The collection of VOCs emitted by beetle-associated microorganisms was conducted 6 days after inoculation of fungi on dishes. Pure cultures of microorganism were grown on PDA dishes, including eight replicates (Petri dishes) for each of the three tested fungal species (i.e., *A. grosmanniae*, *Acremonium* sp., *Cladospoium* sp.), PDA (eight dishes not inoculated with fungi) was included as a negative control. Microbial VOCs were accumulated on self-made passive samplers (PS) obtained from polydimethylsiloxane (PDMS) tubing (Rotilabo silicone tubing, ID 1 mm, OD 1.8 mm, Roth, Karlsruhe, Germany) which were cut into ~5 mm long pieces and washed according to a slightly modified protocol of Kallenbach et al. (2014). Six days after media inoculation, four PS were placed in each dish (including controls) using oven-sterilized metal forceps. Specifically, each group of dishes (including only those of a specific treatment) was first placed inside a sterile working bench. Petri dishes were placed upside down and the PS placed on the dish lid. All dishes were then sealed with Parafilm^®^ for 6 h to allow VOC adsorption on the PS. After sampling, PS were transferred to sterile glass vials, air tight closed and stored at −20°C until analysis.

As for VOCs of beetle associated microorganisms growing *in vitro*, volatiles related to wood pre-infestation or pre-infection were collected by means of PS. Four PS (see above) were placed in individual arenas containing the odor sources tested during the behavioral bioassays, namely: (i) a non-infested beech bolt; (ii) a beech bolt pre-infested by *X. germanus*; (iii) a beech bolt with an artificial hole without fungal inoculation; and (iv) a beech bolt pre-infected by the fungal mutualist *A. grosmanniae*. Volatile collections were carried out 6 days after the initial beetle infestation or fungal inoculation in the bolts. The same time intervals were used for healthy (non-drilled) or drilled (artificial hole only) bolts. Each group of sampling chambers was placed inside a sterile working bench and VOCs collected for 6 h as described above. PS were then transferred to sterile glass vials, air tight closed and stored at −20°C until analysis. There were 7–8 replicates per treatment (volatile source).

For analysis of volatiles by gas-chromatography mass spectrometry (GC–MS), the PS were transferred into glass thermodesorption tubes (Gerstel, Mülheim, Germany). The GC–MS (GC 7890C, MSD 5975C, Agilent, Waldbronn, Germany) was equipped with a thermodesorption/cold-injection system (TDU/CIS, Gerstel, Mülheim, Germany). Thermodesorption tubes containing the PS were heated (220°C) to release the volatile compounds, which were channeled into the CIS for cryo-focusing at −70°C. After complete thermodesorption, the CIS was heated up to 240°C to release the VOCs directly onto the separation column (DB-5 ms ultra-inert, 60 m, Agilent) at a helium flow of 1 mL min^−1^. Oven temperature program and MS settings are described elsewhere (Kleiber et al., 2017). Raw data were processed with the Agilent MassHunter Software (Agilent, Waldbronn, Germany). For tentative compound identification mass spectra of unknown compounds were compared with the MS spectral databank of NIST (National Institute of Standards and Technology, Gaithersburg, MD, United States).

### 2.7. Statistical analysis

Preference behavior of *X. germanus* exhibited during the bioassays was analyzed using Chi-squared goodness-of fit tests to assess whether the beetle response to different sources was significantly different from a 50:50 distribution. Statistical analyses were carried out using SPSS 22.0 software (IBM Corp., Armonk, NY, United States).

To test for differences in the release of volatiles from the different fungi grown on Petri dishes and treatments of beech with beetles and fungus, we tested the data for normal distribution (Shapiro-Wilkinson test). Since normal distribution was not given for each compound, we performed a Kruskal-Wallis test followed by the Dunn test at *p* < 0.05 for VOCs released by wood samples (see Table 1) and a Mann–Whitney test for VOCs released by fungi grown on Petri dishes using peak areas of individual compounds in each sample. Moreover, we applied the web-based software tool “metaboanalyst 5.0” (Xia et al., 2009; Pang et al., 2021) to compare patterns of volatiles from the same samples with a principal component analysis (PCA) after log transformation of raw data.

**Table 1:** MVOCs emitted from fungi isolated from *X. germanus* and grown for 6 days on Petri dishes.

Compound	Match	RT	*A. grosmanniae*	*Acremonium*	*Cladosporium*
	[%]	[min]	Rel. Abundance [EIC/6 h]		
Esters					
Acetic acid propyl ester	91.6	17.03	17 ± 4^a^	3.2 ± 0.3^b^	3.9 ± 1.0^b^
Propanoic acid, 2-methyl-, ethyl ester	93.6	19.56	5.6 ± 1.2		
Acetic acid, 2-methylpropyl ester	96.3	20.41	24 ± 5		
Butanoic acid, 3-methyl-, methyl ester	97.6	20.54	5.9 ± 1.9		
Butanoic acid, ethyl ester	97.5	21.86	36 ± 8		
2-Butenoic acid, 3-methyl-, methyl ester	95.7	24.61	0.3 ± 0.1		
Butanoic acid, 2-methyl-, ethyl ester	98.2	24.77	5.6 ± 1.0^a^	0.1 ± 0.0^b^	
Butanoic acid, 3-methyl-, ethyl ester	98.1	25.03	23 ± 3^a^	0.2 ± 0.0^b^	
Butanoic acid, 2-methyl-		25.50	5.4 ± 2.7		
1-Butanol, 3-methyl-, acetate	94.5	26.39	17 ± 2		
Pentanoic acid, 3-methyl-, methyl ester	95.7	26.66	6.9 ± 0.8		
Propanoic acid, 2-methyl-, 2-methylpropyl ester	94.4	28.41	1.9 ± 0.2		
2-Butenic acid, 2-methyl-, ethyl ester	92.4	30.02	0.8 ± 0.1^a^	0.0 ± 0.0^b^	0.1 ± 0.1^b^
Propanoic acid, 2-hydroxy-, butyl ester		30.42	2.6 ± 0.8^a^		0.0 ± 0.0^b^
Butanoic acid, 3-methyl-, propyl ester	97.3	30.55	2.1 ± 0.9		
Pentanoic acid, 2-hydroxy-4-methyl-, methyl ester		30.76	12 ± 3		
Propanoic acid, pentyl ester		31.51	7.5 ± 0.9^a^		0.1 ± 0.2^b^
Butanoic acid, 2-methyl-, 2-methylpropyl ester	74.5	33.34	0.3 ± 0.1	0.1 ± 0.1	
Butanoic acid, 3-methyl-, 2-methylpropyl ester	97.2	33.51	7.4 ± 0.6		
Isobutyl isopentanoic acid ester	96.0	33.83	5.8 ± 0.5		
Benzoic acid, methyl ester	99.0	37.85	223 ± 48		
Benzoic acid, ethyl ester		40.58	48 ± 3^a^	0.2 ± 0.0^b^	
Acetic acid, 2-phenylethyl ester		43.27	7.4 ± 2.1		
Butanoic acid, 3-methyl-, 3-methyl-3-butenyl ester	96.6	38.34	0.1 ± 0.1		
2-Methyl-2-butenoic acid, isopentyl ester	85.6	40.71	0.2 ± 0.0		
Methyl Phenylacetate	98.6	40.63	10 ± 2		
Decenyl acetate	79.4	47.82		0.6 ± 0.2	
Alcohol					
1-Butanol, 3-methyl-	98.8	18.43	521 ± 96^a^	19 ± 3^b^	
Geosmin	79.9	51.61	0.1 ± 0.0	0.0 ± 0.0	
Globulol	88.4	51.52		7.9 ± 2.1	
Monoterpenoid					
α-Pinene	98.7	30.11	5.7 ± 10.3	7 ± 18	4.0 ± 6.6
β-Pinene	94.0	32.59		2.2 ± 0.6	
Limonene	96.6	35.02		1.7 ± 0.4	
β-Phellandrene	96.0	35.14		6.6 ± 1.0^a^	0.1 ± 0.1^b^
Verbenol	90.9	39.72	0.5 ± 0.9	0.2 ± 0.3	0.1 ± 0.1
Verbenone	95.9	41.96	1.1 ± 2.3		
Limonene oxide	77.1	48.76		0.2 ± 0.0	
Sesquiterpenoid					
Sativene	81.8	45.62	0.1 ± 0.0^b^	4.2 ± 0.3^a^	0.04 ± 0.02^b^
Isolongifolene	90.5	46.30	0.7 ± 0.4^b^	49 ± 5^a^	0.1 ± 0.0^b^
β-Caryophyllene	96.7	48.04		118 ± 5	
α-Chamigrene	87.2	48.35		0.9 ± 0.2^a^	0.0 ± 0.0^b^
α-Copaene	76.3	52.54	0.1 ± 0.5	0.1 ± 0.1	0.0 ± 0.1
Widdrol	75.0	51.83		0.2 ± 0.1	
Caryophyllene oxide	76.3	49.05		0.4 ± 0.1	
Others					
2-deuterio-6-ethoxycarbonylpurine	81.4	48.04	0.7 ± 0.2		
Dodecane, 2,6,11-trimethyl-	95.0	43.35		16.0 ± 2.0	7 ± 8
Benzaldehyde, 2,5-dimethyl-	73.2	47.61		4.4 ± 0.8	

Means ± SD of 7–8 replicates for each fungus are indicated. Significant differences in MVOCs emitted from the different fungal species were calculated with the Kruskal-Wallis test followed by the Dunns test. Differences of VOC release are indicated bold; differences between fungi are indicated by different letters within a row (details on statistical analysis are given in Supplementary Table S1).

## 3. Results

### 3.1. Beetle associated microorganisms

Two different fungal morphotypes were consistently found in association with *X. germanus* dispersing females (see Supplementary Figure S5 in the electronic Supplementary material). Among them, the most commonly occurring fungus was the primary beetle mutualist *A. grosmanniae* that was isolated from all sampled individuals and occurred in 100% of YEMA plates (replicates, *n* = 63). Moreover, *A. grosmanniae* CFUs were the most common occurring among total fungal CFUs in all samples [90.20 ± 2.33%; average ± S.E. of CFUs (%)]. ITS sequence of *A. grosmanniae* isolated in this study (Accession number: OQ513932) showed 100% identity with two *A. grosmanniae* isolates (MG031179.1 and MK118925.1) obtained from *X. germanus* in Europe and the US, respectively (Van de Peppel et al., 2018; Skelton et al., 2019).

Colonies of *Acremonium* sp., the second most prevalent fungal species, were isolated from 47.62% of the total sampled dispersing beetle females. CFU counts showed that this fungus represented 7.72 ± 2.08% of the total fungal colonies occurring among replicates. The *Acremonium* sp. isolated in this study (Accession number: OQ513933) showed 99.24% identity to an undescribed *Acremonium* sp. (KU961664.1) commonly associated with another *Xylosandrus* species (i.e., *X. compactus*) (Bateman et al., 2016). Four others fungal morphotypes were rarely found among replicates, occurring in less than 10% of the tested females and with less than 2% relative abundance (i.e., not-identified Fungus_2: 0.91 ± 0.44%; *Cladosporium* sp.: 0.46 ± 0.32%; not-identified Fungus_5: 0.46 ± 0.32%; not-identified Fungus_6: 0.24 ± 0.24%), suggesting their occasional occurrence as contaminants. Among them, only one fungal species was molecularly characterized as a *Cladosporium* sp. (Accession number: OQ513934) and tested as a representative antagonist in behavioral bioassays. Bacteria and yeasts occurred in 23.81 and 52.38% of replicates, respectively. In particular, an average of 1.14 ± 0.50 CFUs and 47.05 ± 11.58 CFUs was recorded among replicates, for bacteria and yeasts, respectively.

### 3.2. Beetle response to fungal VOCs

**Figure 1:**
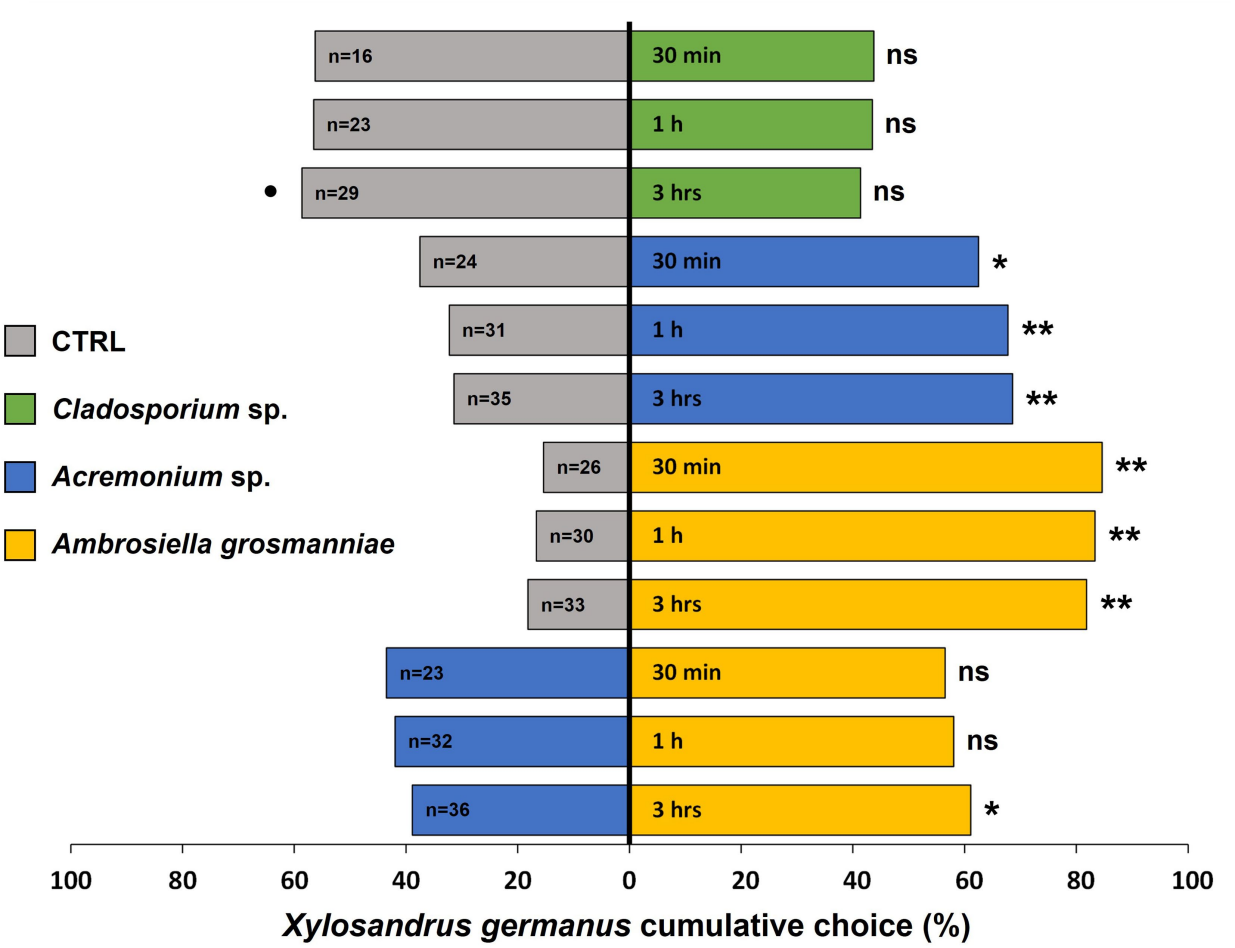
*Xylosandrus germanus* response to volatiles emitted by: (i) its main nutritional symbiont *Ambrosiella grosmanniae* (most common fungus in our study), indicated by yellow bars; (ii) *Acremonium* sp. (the second fungal species consistently found in association with dispersing females in our study), indicated by blue bars; (iii) *Cladosporium* sp. (tested as representative non-symbiont), indicated by green bars; and (iv) PDA control, indicated by grey bars. A total of 40 beetle females were tested for each combination of volatile sources. The choice between different sources was monitored at 30 min, 1 h, and 3 h. Asterisks denote significant differences: *p < 0.05; **p < 0.001. ns, not significant. •*p* < 0.1. *n*, number of insects choosing a volatile source.

*Xylosandrus germanus* females showed a significant preference for MVOCs emitted by the main beetle fungal mutualist *A. grosmanniae* when it was tested against control (pure PDA) at all observation time intervals (30 min: *χ*^2^_1_ = 47.88, *p* < 0.001; 1 h: *χ*^2^_1_ = 44.36, *p* < 0.001; 3 h: *χ*^2^_1_ = 40.45, *p* < 0.001) (Figure 1). Similarly, significantly more beetles orientated toward volatiles emitted by the second most commonly occurring beetle associated fungus *Acremonium* sp. compared to the PDA control (30 min: *χ*^2^_1_ = 6.25, *p* = 0.012; 1 h: *χ*^2^_1_ = 12.53, *p* < 0.001; 3 h: *χ*^2^_1_ = 13.84, *p* < 0.001). By contrast, *X. germanus* individuals did not show any preference, but a trend for repellence was observed after 3 h (30 min: *χ*^2^_1_ = 1.56, *p* = 0.211; 1 h: *χ*^2^_1_ = 1.69, *p* = 0.194; 3 h: *χ*^2^_1_ = 2.96, *p* = 0.085) for the volatiles emitted by the *Cladosporium* sp. compared to those of the PDA control (Figure 1). When the dual choice was between MVOCs emitted by the nutritional symbiont *A. grosmanniae* and *Acremonium* sp., beetles exhibited a significant preference for the main cultivar after 3 h of exposure (*χ*^2^_1_ = 4.94, *p* = 0.026), but not at 30 min (*χ*^2^_1_ = 1.70, *p* = 0.192) and 1 h (*χ*^2^_1_ = 2.61, *p* = 0.107) (Figure 1).

### 3.3. Beetle response to wood pre-infestation or pre-infection

**Figure 2:**
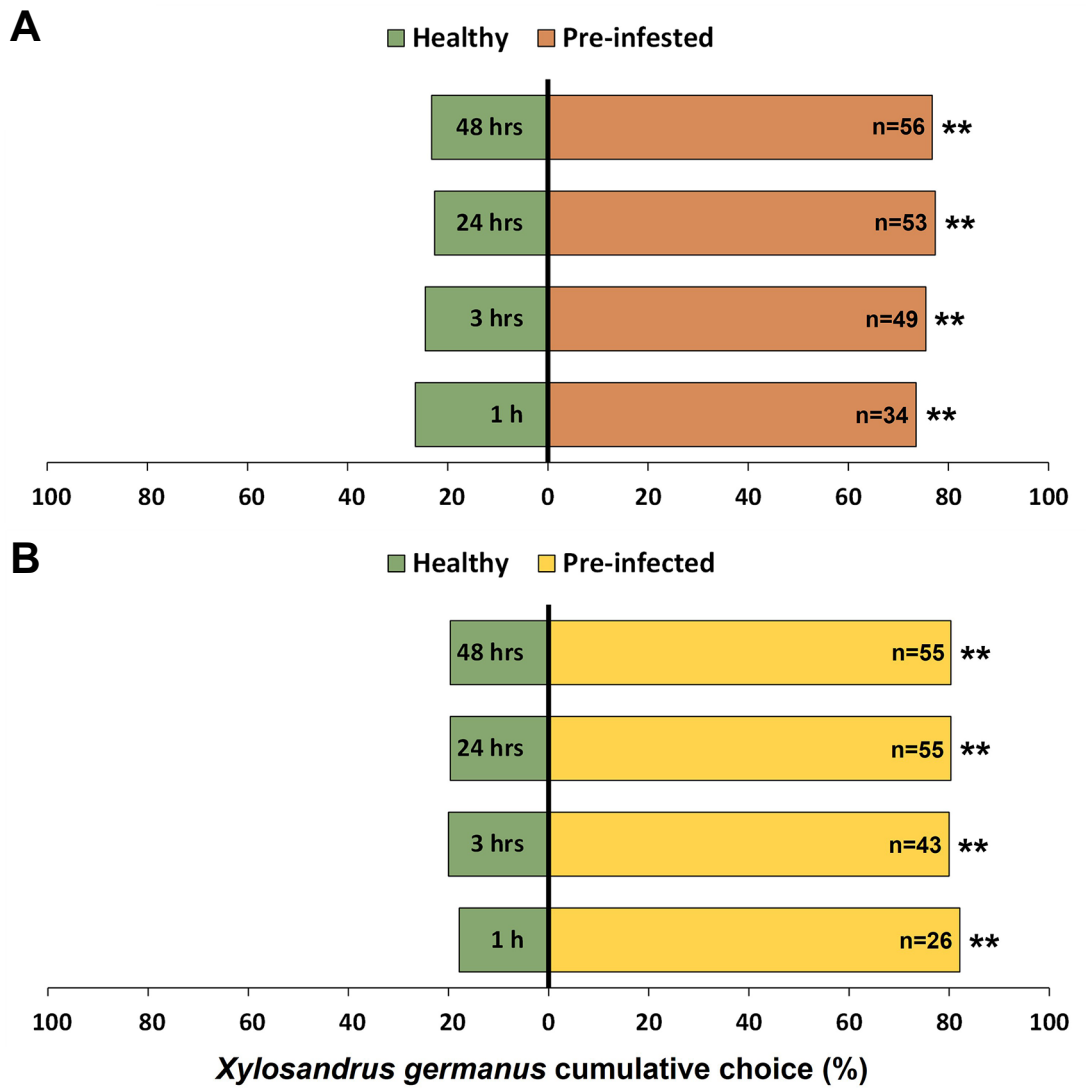
Effects of wood treatment on host choice of *Xylosandrus germanus* adult females. **(A)** Choice assays between previously infested bolts (six days earlier, by a foundress of the same species), indicated by orange bars, and untreated controls, indicated by green bars. **(B)** Choice assays between previously infected bolts (six days earlier, by the main fungal mutualist *A. grosmanniae*), indicated by yellow bars, and non-inoculated controls, indicated by green bars. A total of 60 beetle females were tested and the choice, as measured by tunneling into one of the two bolts (pre-infested or pre-infected vs. control), was monitored at 1 h, 3 h, 24 h, and 48 h. Asterisks denote significant differences: **p < 0.001. *n*, number of insects choosing a source.

*Xylosandrus germanus* significantly preferred to initiate tunneling into bolts previously infested by a conspecific foundress compared to non-infested bolts from the same individual plant (Figure 2A). In particular, significant attraction by beetles for pre-infested bolts was observed at all observation time intervals (1 h: *χ*^2^_1_ = 22.09, *p* < 0.001; 3 h: *χ*^2^_1_ = 26.01, *p* < 0.001; 24 h: *χ*^2^_1_ = 30.03, *p* < 0.001; 48 h: *χ*^2^_1_ = 28.73, *p* < 0.001). Similarly, adults exhibited significant preference for bolts pre-infected with *A. grosmanniae* (Figure 2B) compared to non-inoculated bolts at 1 h (*χ*^2^_1_ = 41.32, *p* < 0.001), 3 h (*χ*^2^_1_ = 36.00, *p* < 0.001), 24 h (*χ*^2^_1_ = 36.87, *p* < 0.001), and 48 h (*χ*^2^_1_ = 36.87, *p* < 0.001).

### 3.4. Microbial VOCs

More than 40 different MVOCs were detected in the emissions from the three fungi isolated from *X. germanus* (Table 1). Esters represented the largest functional group of compounds, which strongly contributed to the volatile profile of *A. grosmanniae* but were less abundant in *Acremoniun* and almost absent in the volatile profile of *Cladosporium*. Monoterpenes were detected in emissions from all fungal species studied, but sesquiterpenes were only detected from the *Acremonium* sp.

**Figure 3:**
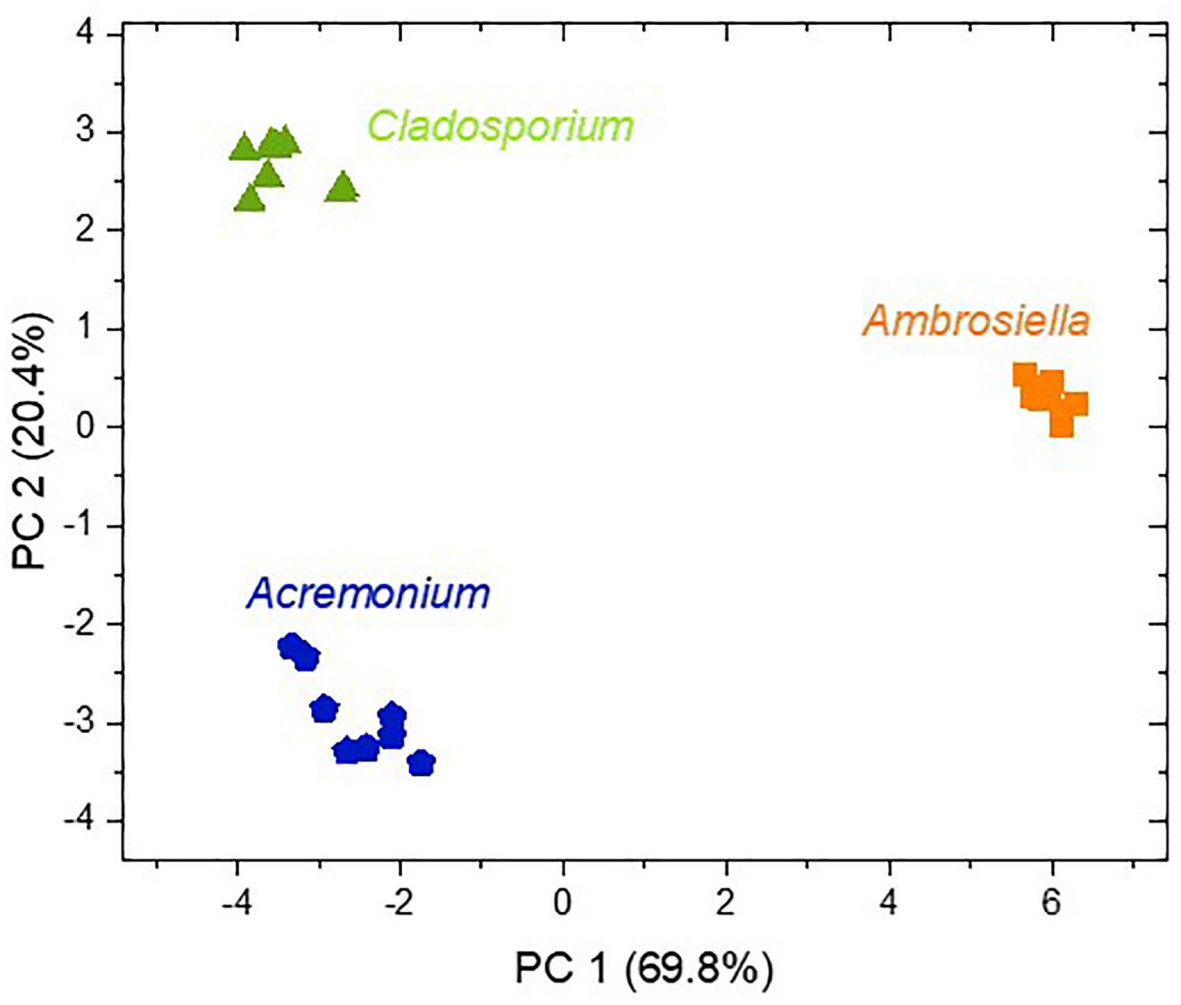
Score plot of a principal component analysis with all MVOCs released from the three fungi isolated from *X. germanus*. The abundances of all MVOC from 7–8 replicates shown in Table 1 were log transformed and used for PCA.

Principal component analysis using all MVOCs detected from the selected fungi revealed distinct clustering by fungal species, hence, underlining characteristic scents of these fungi (Figure 3). PC1 explained to a high degree (70%) separation of *A. grosmanniae* from the *Acremonium* sp. and the *Cladosporium* sp., whereas PC2 rather explained separation of the *Acremonium* sp. from the *Cladosporium* sp. (20%). Separation of *A. grosmanniae* from the other two fungi was mainly driven by differences in the abundance of ester compounds (data not shown).

More than 50 volatile compounds were detected from beech bolts (Table 2). Esters contribute to a large portion of these VOCs (14 different esters), many of them are derivatives of butanoic or propanoic acids. More than 20 alkanes, mainly consisting of C_10_ to C_15_ compounds, were detected from beech bolts. Whereas most of these alkanes were emitted from all bolts independent on the treatment, the esters were mainly observed in beech bolts pre-infested by *X. germanus* or pre-infected with *A. grosmanniae*.

**Table 2:** Volatile compounds emitted from beech bolts infested by *X. germanus* (“beetle”) or infected by *A. grosmanniae* (“fungus”).

Compound	Match	RT	*Beetle*	*Fungus*	*Control*	*Hole*
	[%]	[min]	Rel. Abundance [EIC/6 h]			
Esters						
Propionic acid, 2-methyl-, ethyl ester	93.6	19.44		**49 ± 33**		
Acetic acid, 2-methylpropyl ester	96.3	20.30	0.1 ± 0.3	**35 ± 15**	0.1 ± 0.1	
Butanoic acid, ethyl ester	97.5	21.86		**13 ± 7**		
Butanoic acid, 2-methyl-, ethyl ester	95.1	24.76	**0.3 ± 0.3**	**43 ± 27**	0.7 ± 0.6	0.5 ± 0.1
Butanoic acid, 3-methyl-, ethyl ester	96.3	25.02	**0.0 ± 0.0**	**56 ± 32**	1.4 ± 1.5	
Formic acid, 1,1-dimethylethyl ester	84.9	25.62	**66 ± 112**	70 ± 150	5 ± 2	63 ± 81
1-Butanol, 3-methyl-, acetate	94.5	26.39	**1.4 ± 0.6**	**443 ± 147**	3.7 ± 0.5	
Propanoic acid, 2-methyl-, 2-methylpropyl ester	94.4	28.56		**16 ± 7**		
2-Butenic acid, 2-methyl-, ethyl ester	92.4	30.02		**1.7 ± 0.7**		
Butanoic acid, 2-methyl-, 2-methylpropyl ester	74.5	33.34	0.4 ± 0.4	**2.8 ± 1.3**	0.6 ± 0.3	1.4 ± 1.0
Butanoic acid, 3-methyl-, 2-methylpropyl ester	97.2	33.51	3.8 ± 1.7	**25 ± 9**	3.0 ± 0.9	6.2 ± 1.3
2-Propenoic acid, methyl ester	71.1	38.00		**2.3 ± 1.3**		
Butanoic acid, 3-methyl-, 3-methyl-3-butenyl ester	96.6	38.38	2.6 ± 1.4	0.9 ± 0.6	0.5 ± 0.2	0.7 ± 0.4
Alkanes						
1-Hexene, 3,4-dimethyl	83.0	23.68	**58 ± 47**	8 ± 6	9 ± 8	5 ± 3
Octane, 2,6-dimethyl-	83.4	30.83	**39 ± 22**	29 ± 16	5.6 ± 2.2	39 ± 47
Nonane, 2-methyl-	96.4	31.39	**1.6 ± 4.0**	**2.1 ± 1.5**	0.9 ± 0.2	4.9 ± 1.7
Decane, 5-methyl-	96.2	33.88	**0.6 ± 0.2**	**7.0 ± 1.9**	2.8 ± 0.4	14 ± 4
Nonane, 2,5-dimethyl-	93.3	34.14	**0.4 ± 0.1**	**6.4 ± 1.9**	1.7 ± 0.5	11 ± 4
Nonane, 2,6-dimethyl-	97.8	34.32	**1.1 ± 0.2**	**9.8 ± 1.8**	4.3 ± 0.6	19 ± 5
Dodecane	95.3	37.69	**4.6 ± 0.9**	21 ± 3	5.8 ± 1.0	25 ± 7
Undecane, 2-methyl-	96.0	40.02	**0.2 ± 0.3**	**5.3 ± 0.7**	1.4 ± 0.3	7.1 ± 2.0
Heptadecane,2,5,10,14-tetramethyl	91.0	40.36	7.7 ± 3.3	11 ± 1	4.2 ± 0.9	15 ± 5
Undecane, 2,4-dimethyl-	87.7	41.47	2.1 ± 0.9	4.0 ± 0.6	1.4 ± 0.3	4.8 ± 1.4
Undecane, 2,6-dimethyl-	96.6	41.56	**1.9 ± 0.3**	17 ± 2	4.3 ± 0.9	21 ± 6
Undecane, 4,8-dimethyl-	97.6	41.83	**0.2 ± 0.1**	4.7 ± 0.8	1.2 ± 0.3	6.2 ± 1.9
Hexadecane	96.1	42.39	1.9 ± 0.5	8.7 ± 1.3	2.5 ± 0.4	10 ± 4
Dodecane, 4,6-dimethyl-	97.0	42.73	**0.4 ± 0.1**	12 ± 2	2.6 ± 0.8	13 ± 5
Undecane, 4-ethyl-	90.6	42.98	**2.6 ± 1.3**	21 ± 4	4.8 ± 0.9	22 ± 6
Dodecane, 2,6,11-trimethyl-	95.0	43.35	8.1 ± 0.8	27 ± 4	8.6 ± 1.6	31 ± 11
Dodecane, 2,7,10-trimethyl-	94.1	43.58	1.8 ± 0.2	6.9 ± 0.9	1.7 ± 0.4	7.1 ± 2.8
1-Nonene, 4,6,8-trimethyl	87.6	44.25	**15 ± 3**	4.3 ± 2.1	2.6 ± 0.7	4.0 ± 0.7
Eicosane	91.3	44.74	**3.8 ± 0.5**	3.3 ± 0.6	0.8 ± 0.2	2.8 ± 0.9
Undecane, 4,7-dimethyl-	89.9	44.96	**6.2 ± 1.1**	2.5 ± 0.5	0.6 ± 0.1	2.4 ± 0.7
Octadecane	91.7	48.08	**2.6 ± 0.3**	5.6 ± 1.2	1.8 ± 0.2	6.1 ± 2.1
Octadecane, 2-methyl-		49.43	**11 ± 5**	2.3 ± 0.7	1.5 ± 1.2	2.4 ± 1.4
Oxygenated volatiles						
7-Octen-4-ol	96.3	32.28	6.2 ± 4.4	**3.9 ± 0.8**	3.4 ± 0.6	19 ± 4
1-Hexanol, 2-ethyl-	96.3	34.67	**5.8 ± 2.8**	8.4 ± 3.9	3.0 ± 0.4	8.9 ± 1.8
Octadecane 1-(ethenyloxy)-	84.0	44.03	**18 ± 5**	6.8 ± 0.5	3.7 ± 1.0	8.3 ± 3.9
(+)-1(S)-acetoxy-7(S)-[(benzyloxy)methyl]-6(R)-2-hydroxyprop-2-yl)-7a(R)-hexahydro-3H-pyrrolizin-3-one		48.39	**6.7 ± 2.8**	0.8 ± 0.3	0.5 ± 0.3	1.0 ± 0.6
Phenol, 2,6-bis(1,1-dimethylethyl)-4-methyl-	95.2	48.56	**57 ± 11**	**20 ± 6**	2.0 ± 1.4	3.4 ± 2.0
Others						
α-Pinene	98.7	30.11	**0.9 ± 0.2**	**1.2 ± 0.1**	0.4 ± 0.1	7.7 ± 12.4
Diethyl4,4-dicyano-2,6-di(methoxyimino)-1,7-heptadioate		49.07	**13 ± 4**	3.9 ± 1.0	2.5 ± 2.3	3.6 ± 1.0
2H-2,4a-Ethanonaphthalene, 1,3,4,5,6,7-hexahydro-2,5,5-trimethyl-	89.1	46.30	**0.5 ± 0.1**	**0.2 ± 0.0**	1.3 ± 0.3	20 ± 8

Means ± SD of 7–8 replicates for each treatment are indicated. Statistical significant differences of VOC abundances between beetle infested beech and control beech (“control”) as well as between fungus infected beech and beech with artificial hole (“hole”) were calculated by Mann–Whitney-tests at *p* = 0.05 and are indicated bold (for statistics see also Supplementary Table S2).

**Figure 4:**
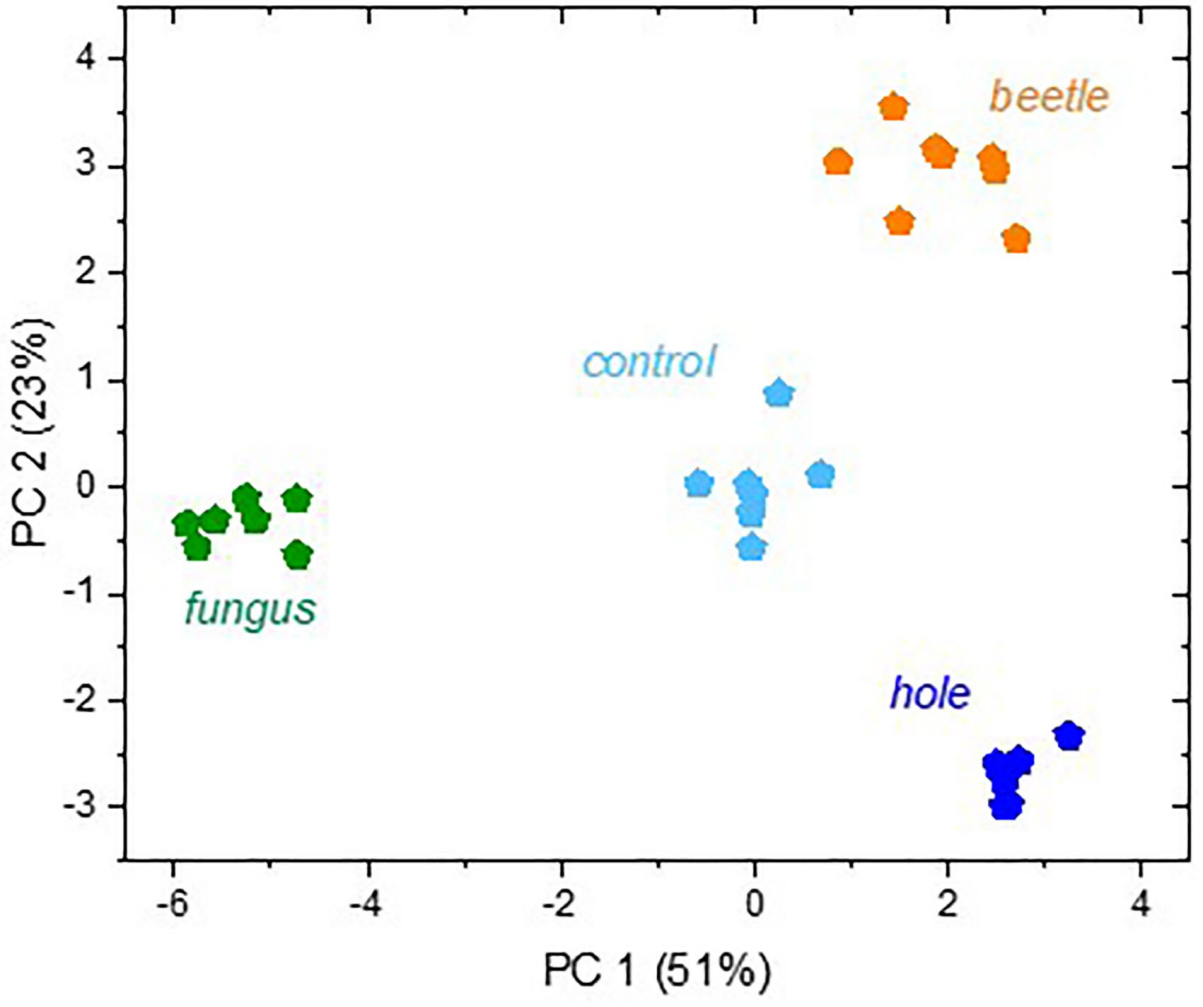
Score plot of a principal component analysis with all VOCs released from the beech bolts. Branch sections were either non-infested (“control”) or infested by *X. germanus* (“beetle”), or they were treated with an artificial hole without infection (“hole”) or with *A. grosmanniae* (“fungus”) infection. The abundances of all VOC from 7–8 replicates shown in Table 2 were log transformed and used for PCA.

Multivariate analysis indicated distinct volatile emissions from the different beech bolts (Figure 4). Principal component 1 (PC1) explained to a high degree (51%) separation of *A. grosmanniae* infected bolts from its control (bolts with artificial hole) and the other treatments, whereas PC2 rather described the separation of the *X. germanus* infested bolts from its control and the remainder variants (23%).

**Figure 5:**
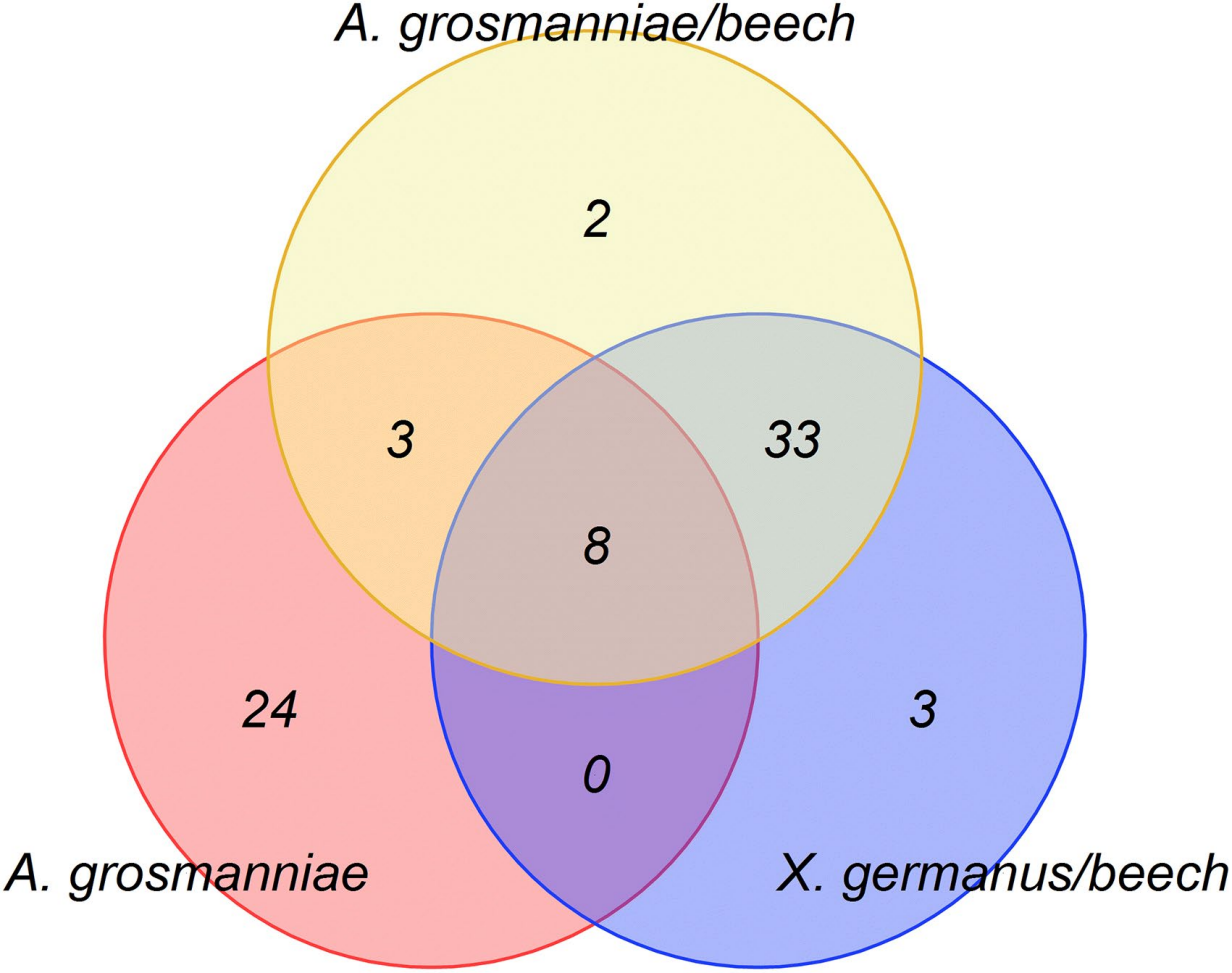
Venn Diagram indicating the volatile organic compounds commonly emitted from the different samples [i.e., beech bolts infested by *X. germanus* (“*X. germanus*/beech”); beech bolts infected by *A. grosmanniae* (“*A. grosmanniae*/beech”); *A. grosmanniae* growing *in vitro* (“*A. grosmanniae*”)].

The bioassays indicated attractance of ambrosia beetles to the scents, which were released by *A. grosmanniae* (Figure 1), and by beech bolts either infected by *A. grosmanniae* or infested by *X. germanus* (Figure 2). To obtain hints on the volatiles responsible for the luring effect, we generated a Venn diagram including all volatiles released by attractive samples (Figure 5). This diagram indicates a relatively high degree of overlap (11 compounds) between *A. grosmanniae* grown in Petri dishes and beech bolts infected by this fungus. Most of these VOCs were ester compounds (Tables 1, 2). An even higher overlap (41 compounds) was found for the beech bolt infested by *X. germanus* and infected by *A. grosmanniae*. Most of the overlapping compounds were alkanes as also indicated by Table 2. Eight VOCs were commonly released from beech bolts infested by *X. germanus*, beech bolts infected by *A. grosmanniae*, and *A. grosmanniae* growing on PDA, namely, (i) 1-butanol, 3-methyl-, acetate (RT 26.30), (ii) acetic acid, 2-methylpropyl ester (RT 20.30), (iii) butanoic acid, 3-methyl-, 3-methyl-3-butenyl ester (RT 38.38), (iv) butanoic acid, 2-methyl, −ethyl ester (RT 24.76), (v) butanoic acid, 3-methyl, −ethyl ester (RT 25.02), (vi) butanoic acid, 2-methyl-, 2-methylpropyl ester (RT 33.34), and (vii) butanoic acid, 3-methyl-, 2-methylpropyl ester (RT 33.51), and (viii) α-pinene (RT 30.11).

## 4. Discussion

Ethanol has been extensively demonstrated to act as a long- and short-range attractant for *X. germanus* and other ambrosia beetles, but the role of other species-specific chemicals eliciting behavioral responses in dispersing individuals remains poorly investigated (Ranger et al., 2021b). In particular, the influence of volatiles from symbiotic and auxiliary microorganisms on the behavior of fungus-farming ambrosia beetles is of growing interest (Cale et al., 2016; Kandasamy et al., 2019; Kendra et al., 2022). During still-air walking bioassays, Ranger et al. (2021b) observed a stronger arrestment response of adult female *X. germanus* to volatiles of its nutritional fungal symbiont (growing *in vitro*) compared to antagonistic fungi. In our current study, we report the ability of *X. germanus* to discriminate between volatiles emitted by different associated fungi, orientating its choice according to the different combinations of odorous sources. More importantly, our results show, for the first time, the potential of MVOCs produced by the main beetle nutritional symbiont *A. grosmanniae*, growing in wood (*in vivo*), to significantly affect beetles’ orientation behavior and brood site selection. This strongly suggests that adult beetles use these fungal cues for mass aggregation on certain tree hosts and it is thus the first evidence for an aggregation pheromone in a xyleborine ambrosia beetle.

The composition of the microbial community associated with dispersing *X. germanus* individuals in the present study revealed a strong dominance of two fungal species in addition to unknown bacteria and yeasts. These species, *A. grosmanniae* and *Acremonium* sp., were isolated from 100 and 48% of sampled beetle individuals, respectively. However, while the nutritional role of the *X. germanus* species-specific mutualistic fungus *A. grosmanniae* has been extensively described (Mayers et al., 2015; van de Peppel et al., 2018), the role of the common *Acremonium* species remains to be clarified. Symbionts in the genus *Acremonium* have been repeatedly reported from *Xylosandrus* spp. (Bateman et al., 2016; Gugliuzzo et al., 2020). For example, one strain was isolated from *X. germanus* mycetangia (26% frequency) and body parts (33% frequency) in Turkey (Tuncer et al., 2017). Another *Acremonium* species (identified as *A. kiliense* by the authors) was found in association with *X. germanus* infested galleries in China, representing one of the most commonly occurring fungal species together with *Ambrosiella hartigii* and *Fusarium* spp. (Yang et al., 2008). Since *Acremonium* species possess carbohydrate-active enzymes capable of degrading lignocellulose components (Lopes et al., 2020), their association with *X. germanus* could potentially facilitate the degradation of host tree tissues during colonization. Bacteria and yeasts have been also isolated from *X. germanus* individuals or infested galleries, but their functions have not been determined (Hulcr et al., 2012; Agnello et al., 2017; Rassati et al., 2019). In some cases, bacteria found in association with ambrosia beetles have been considered as beneficial (Haanstad and Norris, 1985; Grubbs et al., 2011, 2020; Barcoto et al., 2020; Diehl et al., 2022). Further studies are needed to characterize the specific ecological role of all these microorganisms for *X. germanus*.

Results of dual-choice bioassays revealed a strong ability by *X. germanus* females to discriminate between VOCs emitted by commonly beetle associated fungi (*A. grosmanniae* and *Acremonium* sp.) and those produced by a representative non-symbiont or putative antagonist (*Cladosporium* sp.) (Figure 1). In particular, the beetle orientation behavior was significantly affected by fungal VOCs, with *X. germanus* females exhibiting significant attraction for volatiles of the nutritional symbiont rather than those of the control (only PDA), corroborating results obtained by Ranger et al. (2021a). Moreover, our results highlighted that beetles can discriminate between different microbial sources (i.e., MVOCs of different fungi). However, *X. germanus* exhibited positive response to *A. grosmanniae* volatiles, when compared to those of *Acremonium* sp., only after 3 h of observation, and not after 30 min or 1 h. This result suggests that MVOCs of both associated fungi are attractive for dispersing females but that the beetle is able to discriminate among them only after long exposure time.

The beetle’s positive orientation toward VOCs produced by the main fungal mutualist *A. grosmanniae* was confirmed during dual choice bioassays with pre-infected or pre-infested wood. Indeed, a clear preference by *X. germanus* for VOCs of bolts already inoculated with *A. grosmanniae* or infested by a conspecific foundress, compared to healthy ones, was observed. In both bioassays the beetle’s nutritional symbiont occurred in bolts (pre-infected or pre-infested) either artificially or naturally (by the beetle) inoculated (see Supplementary Figure S3). In the first bioassay, beetle females consistently chose to start infesting the already inoculated beech bolt (Figure 2B), confirming their attraction to the mutualistic fungus. In the second bioassay, beetle females significantly preferred to colonize bolts already infested by conspecific foundresses, suggesting the potential of fungal VOCs to act as aggregation cues.

To the best of our knowledge, this is the first study investigating the role of MVOCs emitted by a mutualistic fungus growing *in vivo* (wood tissues) as potential aggregation source for ambrosia beetles in the genus *Xylosandrus*. Pheromone production has not been documented for ambrosia beetles in the tribe Xyleborini (Kirkendall, 1997), however heavy *Xylosandrus* infestation followed by aggregation of several individuals in specific parts of the host tree have been often reported (Gallego et al., 2017; Hulcr and Stelinski, 2017; Gugliuzzo et al., 2019, 2021). Based on our results, it is possible to speculate that MVOCs produced or elicited by *X. germanus* associated fungi during tree infestation by foundresses (which located the stressed host by means of ethanol) can be detected by other host-seeking females, which recognize the suitable woody tissues and consequently tend to colonize the same host site, excavating their own galleries and aggregating in it. In such a scenario, ethanol could act as a long-range attractant for dispersing (i.e., flying) *X. germanus* females, which may use then specific MVOCs for short-range orientation on the bolts.

Our volatile collections and GC–MS analyses detected eight MVOCs that were consistently released from beech bolts infested by *X. germanus* or infected by *A. grosmanniae*, and *A. grosmanniae* growing on PDA. Additional studies are warranted to determine if these MVOCs, individually or blends, influence the long- and/or short-range attraction of *X. germanus* to host tissues (Figure 5). Compared to our study, Ranger et al. (2021a) detected lower molecular weight MVOCs in emissions from *A. grosmanniae* growing on malt extract agar, specifically, 2-ethyl-1-hexanol, 2-phenylethanol, methyl benzoate, and 3-methyl-1-butanol. Female *X. germanus* exhibited a weak or negligible response to these individual volatiles during walking bioassays. Since *X. germanus* exhibited an arrestment response to cultures of *A. grosmanniae* growing on MEA, blends of the MVOCs emitted from *A. grosmanniae* are potentially more behaviorally informative than individual compounds.

From an evolutionary perspective is not obvious why MVOCs of fungal symbionts should be used as aggregation cues for beetles colonizing dead wood, because bark beetles are generally known to strongly suffer from intraspecific competition (Kirkendall et al., 2015) and thus beetles should avoid to breed near others. Furthermore, females reliably transmit their cultivars in mycetangia to new hosts and so additional horizontal acquisition of foreign strains may actually weaken the mutualism (Frank, 1994, 1996; Sachs and Simms, 2006). So, what could be the benefit of being attracted to a host that does not need to be overwhelmed (like in tree-killing bark beetles)? We hypothesize that MVOCs may signal arriving beetles a suitable substrate for growing their delicate and highly substrate-specific fungal cultivars. It has been reported that in xyleborine ambrosia beetles more than 80% of females fail to establish nests, which is mostly caused by unsuitable substrates (Biedermann et al., 2009).

In closing, our current study demonstrates that MVOCs emitted from infested host tissues can influence the attraction of *X. germanus*. Additional studies are warranted to identify the behaviorally active MVOCs and their potential for increasing attraction to ethanol, which is well known to be highly attractive to *X. germanus*. These efforts could potentially lead to the development of semiochemical-based control tactics. Moreover, since MVOCs from the specific primary fungal mutualists were attractive in our current study, a highly targeted and selective attraction of only *X. germanus* may occur. Consequently, these results might aid in the development of selective trapping methods for *X. germanus* that could be integrated into a “push-pull” strategy.

## Data availability statement

The original contributions presented in the study are included in the article, further inquiries can be directed to the corresponding authors. The data (i.e., fungal sequences) presented in the study are deposited in the NCBI GenBank database (https://www.ncbi.nlm.nih.gov) and are publicly available (Accession numbers are included in the Results section 3.1).

## Author contributions

AG and PB conceived the project, designed the experiments, and wrote the manuscript with inputs from all the co-authors. AG and JK performed the experiments and analyzed the data. All authors contributed to the article and approved the submitted version.

## Funding

AG was funded by the German Academic Exchange Service (DAAD Research Grant–57552336) and the University of Catania, Italy (PhD Grant). PB and JK were funded by the University of Freiburg, Germany. PB’s research is funded by the German Research Foundation (DFG) (Emmy Noether grant number BI 1956/1–1). CR was funded by the USDA-Agricultural Research Service, USA. GG and AB were funded by University of Catania, Italy (Projects “Emergent Pests and Pathogens and Relative Sustainable Strategies–5A722192113” and “PIA.CE.RI. 2020-2022 Linea 4 Open Access -5A722192177”). This publication was supported by the Open Access Publication Fund of the University of Freiburg.

## Conflict of interest

The authors declare that the research was conducted in the absence of any commercial or financial relationships that could be construed as a potential conflict of interest.

## Publisher’s note

All claims expressed in this article are solely those of the authors and do not necessarily represent those of their affiliated organizations, or those of the publisher, the editors and the reviewers. Any product that may be evaluated in this article, or claim that may be made by its manufacturer, is not guaranteed or endorsed by the publisher.

## References

[ref1] Agnello A. M. Breth D. I. Tee E. M. Cox K. D. Villani S. M. Ayer K. M.. (2017). *Xylosandrus germanus* (Coleoptera: *Curculionidae*: Scolytinae) occurrence, fungal associations, and management trials in New York apple orchards. J. Econ. Entomol. 110, 2149–2164. doi: 10.1093/jee/tox189, PMID: 29048587

[ref9001] Altschul S. F. Gish W. Miller W. Myers E. W. Lipman D. J. (1990). Basic local alignment search tool. J. Mol. Biol. 215, 403–410. doi: 10.1016/S0022-2836(05)80360-2 2231712

[ref2] Barcoto M. O. Carlos-Shanley C. Fan H. Ferro M. Nagamoto N. S. Bacci M.. (2020). Fungus-growing insects host a distinctive microbiota apparently adapted to the fungiculture environment. Sci. Rep. 10, 1–13. doi: 10.1038/s41598-020-68448-7 32709946 PMC7381635

[ref3] Bateman C. Šigut M. Skelton J. Smith K. E. Hulcr J. (2016). Fungal associates of the *Xylosandrus compactus* (Coleoptera: Curculionidae, Scolytinae) are spatially segregated on the insect body. Environ. Entomol. 45, 883–890. doi: 10.1093/ee/nvw070, PMID: 27357160

[ref9002] Biedermann P. H. Klepzig K. D. Taborsky M. (2009). Fungus cultivation by ambrosia beetles: behavior and laboratory breeding success in three xyleborine species. Environ. Entomol. 38, 1096–1105. doi: 10.1603/022.038.0417 19689888

[ref4] Biedermann P. H. Vega F. E. (2020). Ecology and evolution of insect–fungus mutualisms. Annu. Rev. Entomol. 65, 431–455. doi: 10.1146/annurev-ento-011019-024910, PMID: 31610133

[ref6] Bueno E. Martin K. R. Raguso R. A. Mcmullen J. G. Hesler S. P. Loeb G. M.. (2020). Response of wild spotted wing drosophila (*Drosophila suzukii*) to microbial volatiles. J. Chem. Ecol. 46, 688–698. doi: 10.1007/s10886-019-01139-4, PMID: 31879864

[ref7] Cale J. A. Collignon R. M. Klutsch J. G. Kanekar S. S. Hussain A. Erbilgin N. (2016). Fungal volatiles can act as carbon sources and semiochemicals to mediate interspecific interactions among bark beetle-associated fungal symbionts. PLoS One 11:e0162197. doi: 10.1371/journal.pone.0162197, PMID: 27583519 PMC5008770

[ref8] Carthey A. J. Gillings M. R. Blumstein D. T. (2018). The extended genotype: microbially mediated olfactory communication. Trends Ecol. Evol. 33, 885–894. doi: 10.1016/j.tree.2018.08.010, PMID: 30224089

[ref9] Contarini M. Vannini A. Giarruzzo F. Faccoli M. Morales-Rodriguez C. Rossini L.. (2020). First record of *Xylosandrus germanus* (Blandford) (Coleoptera: Curculionidae, Scolytinae) in the Mediterranean scrubland in southern Italy, and its co-presence with the co-generic species *X. compactus* (Eichhoff) and *X. crassiusculus* (Motschulsky). EPPO Bull 50, 311–315. doi: 10.1111/epp.12660

[ref10] Crowley-Gall A. Rering C. C. Rudolph A. B. Vannette R. L. Beck J. J. (2021). Volatile microbial semiochemicals and insect perception at flowers. Curr. Opin. Insect Sci. 44, 23–34. doi: 10.1016/j.cois.2020.10.004, PMID: 33096275

[ref11] Davis T. S. Crippen T. L. Hofstetter R. W. Tomberlin J. K. (2013). Microbial volatile emissions as insect semiochemicals. J. Chem. Ecol. 39, 840–859. doi: 10.1007/s10886-013-0306-z, PMID: 23793954

[ref12] de Beer Z. W. Procter M. Wingfield M. J. Marincowitz S. Duong T. A. (2022). Generic boundaries in the Ophiostomatales reconsidered and revised. Stud. Mycol. 101, 57–120. doi: 10.3114/sim.2022.101.02, PMID: 36059894 PMC9365045

[ref13] Diehl J. M. Kowallik V. Keller A. Biedermann P. H. (2022). First experimental evidence for active farming in ambrosia beetles and strong heredity of garden microbiomes. Proc. Royal Soc. B. 289:20221458. doi: 10.1098/rspb.2022.1458, PMID: 36321493 PMC9627711

[ref14] Dzurenko M. Ranger C. M. Hulcr J. Galko J. Kaňuch P. (2021). Origin of non-native *Xylosandrus germanus*, an invasive pest ambrosia beetle in Europe and North America. J. Pest. Sci. 94, 553–562. doi: 10.1007/s10340-020-01283-x

[ref15] Egonyu J. P. Torto B. (2018). Responses of the ambrosia beetle *Xylosandrus compactus* (Coleoptera: Curculionidea: Scolytinae) to volatile constituents of its symbiotic fungus *fusarium solani* (Hypocreales: Nectriaceae). Arthropod Plant Interact. 12, 9–20. doi: 10.1007/s11829-017-9552-2

[ref16] Engl T. Kaltenpoth M. (2018). Influence of microbial symbionts on insect pheromones. Nat. Prod. Rep. 35, 386–397. doi: 10.1039/C7NP00068E, PMID: 29565067

[ref17] Fleischer J. Pregitzer P. Breer H. Krieger J. (2018). Access to the odor world: olfactory receptors and their role for signal transduction in insects. Cell. Mol. Life Sci. 75, 485–508. doi: 10.1007/s00018-017-2627-5, PMID: 28828501 PMC11105692

[ref18] Frank S. A. (1994). Genetics of mutualism: the evolution of altruism between species. J. Theor. Biol. 170, 393–400. doi: 10.1006/jtbi.1994.1200, PMID: 7996864

[ref19] Frank S. A. (1996). Host–symbiont conflict over the mixing of symbiotic lineages. Proc. Royal Soc. Lond. B Biol Sci. 263, 339–344. doi: 10.1098/rspb.1996.0052 8920255

[ref20] Galko J. Dzurenko M. Ranger C. M. Kulfan J. Kula E. Nikolov C.. (2019). Distribution, habitat preference, and management of the invasive ambrosia beetle *Xylosandrus germanus* (Coleoptera: Curculionidae, Scolytinae) in European forests with an emphasis on the West Carpathians. Forests 10:10. doi: 10.3390/f10010010

[ref21] Gallego D. Lencina J. L. Mas H. Ceveró J. Faccoli M. (2017). First record of the granulate ambrosia beetle, *Xylosandrus crassiusculus* (Coleoptera: Curculionidae, Scolytinae), in the Iberian Peninsula. Zootaxa 4273, 431–434. doi: 10.11646/zootaxa.4273.3.7, PMID: 28610243

[ref22] Grubbs K. J. Biedermann P. H. Suen G. Adams S. M. Moeller J. A. Klassen J. L.. (2011). Genome sequence of *Streptomyces griseus* strain Xyleb KG-1, an ambrosia beetle-associated actinomycete. J. Bacteriol. 193, 2890–2891. doi: 10.1128/JB.00330-11, PMID: 21460079 PMC3133108

[ref23] Grubbs K. J. Surup F. Biedermann P. H. McDonald B. R. Klassen J. L. Carlson C. M.. (2020). Cycloheximide-producing Streptomyces associated with *Xyleborinus saxesenii* and *Xyleborus affinis* fungus-farming ambrosia beetles. Front. Microbiol. 11:2207. doi: 10.3389/fmicb.2020.562140 PMC7546818 33101237

[ref24] Gugliuzzo A. Aiello D. Biondi A. Giurdanella G. Siscaro G. Zappalà L.. (2022). Microbial mutualism suppression by *Trichoderma* and *bacillus* species for controlling the invasive ambrosia beetle *Xylosandrus compactus*. Biol. Control 170:104929. doi: 10.1016/j.biocontrol.2022.104929

[ref25] Gugliuzzo A. Biedermann P. H. Carrillo D. Castrillo L. A. Egonyu J. P. Gallego D.. (2021). Recent advances toward the sustainable management of invasive *Xylosandrus* ambrosia beetles. J. Pest. Sci. 94, 615–637. doi: 10.1007/s10340-021-01382-3

[ref26] Gugliuzzo A. Criscione G. Biondi A. Aiello D. Vitale A. Polizzi G.. (2020). Seasonal changes in population structure of the ambrosia beetle *Xylosandrus compactus* and its associated fungi in a southern Mediterranean environment. PLoS One 15:e0239011. doi: 10.1371/journal.pone.0239011, PMID: 32915885 PMC7485756

[ref27] Gugliuzzo A. Criscione G. Tropea Garzia G. (2019). Unusual behavior of *Xylosandrus compactus* (Coleoptera: Scolytinae) on carob trees in a Mediterranean environment. Insects 10:82. doi: 10.3390/insects10030082, PMID: 30909589 PMC6468604

[ref28] Guo H. Wang C. Z. (2019). The ethological significance and olfactory detection of herbivore-induced plant volatiles in interactions of plants, herbivorous insects, and parasitoids. Arthropod Plant Interact. 13, 161–179. doi: 10.1007/s11829-019-09672-5

[ref29] Haanstad J. O. Norris D. (1985). Microbial symbiotes of the ambrosia beetle *Xyloterinus politus*. Microb. Ecol. 11, 267–276. doi: 10.1007/BF02010605, PMID: 24221366

[ref30] Hulcr J. Mann R. Stelinski L. L. (2011). The scent of a partner: ambrosia beetles are attracted to volatiles from their fungal symbionts. J. Chem. Ecol. 37, 1374–1377. doi: 10.1007/s10886-011-0046-x, PMID: 22161224

[ref31] Hulcr J. Rountree N. R. Diamond S. E. Stelinski L. L. Fierer N. Dunn R. R. (2012). Mycangia of ambrosia beetles host communities of bacteria. Microb. Ecol. 64, 784–793. doi: 10.1007/s00248-012-0055-5, PMID: 22546962

[ref32] Hulcr J. Stelinski L. L. (2017). The ambrosia symbiosis: from evolutionary ecology to practical management. Annu. Rev. Entomol. 62, 285–303. doi: 10.1146/annurev-ento-031616-035105, PMID: 27860522

[ref34] Ito M. Kajimura H. (2017). Landscape-scale genetic differentiation of a mycangial fungus associated with the ambrosia beetle, *Xylosandrus germanus* (Blandford) (Curculionidae: Scolytinae) in Japan. Ecol. Evol. 7, 9203–9221. doi: 10.1002/ece3.3437, PMID: 29187962 PMC5696423

[ref35] Kallenbach M. Oh Y. Eilers E. J. Veit D. Baldwin I. T. Schuman M. C. (2014). A robust, simple, high-throughput technique for time-resolved plant volatile analysis in field experiments. Plant J. 78, 1060–1072. doi: 10.1111/tpj.12523, PMID: 24684685 PMC4190661

[ref36] Kandasamy D. Gershenzon J. Andersson M. N. Hammerbacher A. (2019). Volatile organic compounds influence the interaction of the Eurasian spruce bark beetle (*Ips typographus*) with its fungal symbionts. ISME J. 13, 1788–1800. doi: 10.1038/s41396-019-0390-3, PMID: 30872804 PMC6775991

[ref37] Kendra P. E. Tabanca N. Cruz L. F. Menocal O. Schnell E. Q. Carrillo D. (2022). Volatile emissions and relative attraction of the fungal symbionts of tea shot hole borer (Coleoptera: Curculionidae). Biomol. Ther. 12:97. doi: 10.3390/biom12010097, PMID: 35053245 PMC8773808

[ref38] Kirkendall L. R. (1997). “Interactions among males, females and offspring in bark and ambrosia beetles: the significance of living in tunnels for the evolution of social behavior” in The Evolution of Social Behavior in Insects and Arachnids. eds. Choe J. C. Crespi B. J. (Cambridge, UK: Cambridge University Press), 181–215.

[ref9004] Kirkendall L. R. Biedermann P. H. Jordal B. H. (2015). “Evolution and diversity of bark and ambrosia beetles” in Bark beetles. eds. Vega E. Hofstetter R. W. (Academic Press), 85–156.

[ref39] Kleiber A. Duan Q. Jansen K. Verena Junker L. Kammerer B. Rennenberg H.. (2017). Drought effects on root and needle terpenoid content of a coastal and an interior Douglas fir provenance. Tree Physiol. 37, 1648–1658. doi: 10.1093/treephys/tpx113, PMID: 29036462

[ref40] Kuhns E. H. Tribuiani Y. Martini X. Meyer W. L. Peña J. Hulcr J.. (2014). Volatiles from the symbiotic fungus *Raffaelea lauricola* are synergistic with Manuka lures for increased capture of the Redbay ambrosia beetle *Xyleborus glabratus*. Agric. For. Entomol. 16, 87–94. doi: 10.1111/afe.12037

[ref41] Lehenberger M. Foh N. Göttlein A. Six D. Biedermann P. H. (2021). Nutrient-poor breeding substrates of ambrosia beetles are enriched with biologically important elements. Front. Microbiol. 12:927. doi: 10.3389/fmicb.2021.664542 PMC8107399 33981292

[ref43] Lopes A. M. M. de Mélo A. H. F. Procopio D. P. Teixeira G. S. Carazzolle M. F. de Carvalho L. M.. (2020). Genome sequence of *Acremonium strictum* AAJ6 strain isolated from the Cerrado biome in Brazil and CAZymes expression in thermotolerant industrial yeast for ethanol production. Process Biochem. 98, 139–150. doi: 10.1016/j.procbio.2020.07.029

[ref44] Martini X. Hughes M. A. Killiny N. George J. Lapointe S. L. Smith J. A.. (2017). The fungus *Raffaelea lauricola* modifies behavior of its symbiont and vector, the redbay ambrosia beetle (*Xyleborus glabratus*), by altering host plant volatile production. J. Chem. Ecol. 43, 519–531. doi: 10.1007/s10886-017-0843-y, PMID: 28455797

[ref45] Mayers C. G. Harrington T. C. Biedermann P. H. (2022). “Mycangia define the diverse ambrosia beetle–fungus symbioses” in The Convergent Evolution of Agriculture in Humans and Insects. eds. Schultz T. R. Gawne R. Peregrine P. N. (Cambridge, MA: The MIT Press), 105–142.

[ref46] Mayers C. G. McNew D. L. Harrington T. C. Roeper R. A. Fraedrich S. W. Biedermann P. H.. (2015). Three genera in the Ceratocystidaceae are the respective symbionts of three independent lineages of ambrosia beetles with large, complex mycangia. Fungal Biol. 119, 1075–1092. doi: 10.1016/j.funbio.2015.08.002, PMID: 26466881

[ref47] Netherer S. Kandasamy D. Jirosová A. Kalinová B. Schebeck M. Schlyter F. (2021). Interactions among Norway spruce, the bark beetle *Ips typographus* and its fungal symbionts in times of drought. J. Pest. Sci. 94, 591–614. doi: 10.1007/s10340-021-01341-y, PMID: 34720785 PMC8550215

[ref48] Nones S. Sousa E. Holighaus G. (2022). Symbiotic fungi of an ambrosia beetle alter the volatile bouquet of cork oak seedlings. Phytopathology 112, 1965–1978. doi: 10.1094/PHYTO-08-21-0345-R, PMID: 35357159

[ref49] Norin T. (2007). Semiochemicals for insect pest management. Pure Appl. Chem. 79, 2129–2136. doi: 10.1351/pac200779122129

[ref50] Norris D. M. Baker J. M. (1968). A minimal nutritional substrate required by *fusarium solani* to fulfill its mutualistic relationship with *Xyleborus ferrugineus*. Ann. Entomol. Soc. Am. 61, 1473–1475. doi: 10.1093/aesa/61.6.1473

[ref51] Pang Z. Chong J. Zhou G. de Lima Morais D. A. Chang L. Barrette M.. (2021). Metabo analyst 5.0: narrowing the gap between raw spectra and functional insights. Nucleic Acids Res. 49, W388–W396. doi: 10.1093/nar/gkab382 34019663 PMC8265181

[ref52] Peer K. Taborsky M. (2004). Female ambrosia beetles adjust their offspring sex ratio according to outbreeding opportunities for their sons. J. Evol. Biol. 17, 257–264. doi: 10.1111/j.1420-9101.2003.00687.x, PMID: 15009259

[ref53] Rabaglia R. J. Dole S. A. Cognato A. I. (2006). Review of American Xyleborina (Coleoptera: Curculionidae: Scolytinae) occurring north of Mexico, with an illustrated key. Ann. Entomol. Soc. Am. 99, 1034–1056. doi: 10.1603/0013-8746(2006)99[1034:ROAXCC]2.0.CO;2

[ref9003] Ranger C. M. Biedermann P. H. Phuntumart V. Beligala G. U. Ghosh S. Palmquist D. E. (2018). Symbiont selection via alcohol benefits fungus farming by ambrosia beetles. PNAS. 115, 4447–4452. doi: 10.1073/pnas.1716852115 29632193 PMC5924889

[ref54] Ranger C. M. Dzurenko M. Barnett J. Geedi R. Castrillo L. Ethington M.. (2021a). Electrophysiological and behavioral responses of an ambrosia beetle to volatiles of its nutritional fungal symbiont. J. Chem. Ecol. 47, 463–475. doi: 10.1007/s10886-021-01263-0, PMID: 33761047 PMC8116273

[ref55] Ranger C. M. Reding M. E. Addesso K. Ginzel M. Rassati D. (2021b). Semiochemical-mediated host selection by *Xylosandrus* spp. ambrosia beetles (Coleoptera: Curculionidae) attacking horticultural tree crops: a review of basic and applied science. Can. Entomol. 153, 103–120. doi: 10.4039/tce.2020.51

[ref56] Ranger C. M. Reding M. E. Schultz P. B. Oliver J. B. Frank S. D. Addesso K. M.. (2016). Biology, ecology, and management of nonnative ambrosia beetles (Coleoptera: Curculionidae: Scolytinae) in ornamental plant nurseries. J. Integr. Pest Manag. 7:5. doi: 10.1093/jipm/pmw005

[ref57] Rassati D. Marini L. Malacrinò A. (2019). Acquisition of fungi from the environment modifies ambrosia beetle mycobiome during invasion. PeerJ 7:e8103. doi: 10.7717/peerj.8103, PMID: 31763076 PMC6870512

[ref58] Ren L. Ma Y. Xie M. Lu Y. Cheng D. (2021). Rectal bacteria produce sex pheromones in the male oriental fruit fly. Curr. Biol. 31, 2220–2226.e4. doi: 10.1016/j.cub.2021.02.046, PMID: 33740424

[ref59] Sachs J. L. Simms E. L. (2006). Pathways to mutualism breakdown. Trends Ecol. Evol. 21, 585–592. doi: 10.1016/j.tree.2006.06.018 16828927

[ref60] Schmidt H. R. Benton R. (2020). Molecular mechanisms of olfactory detection in insects: beyond receptors. Open Biol. 10:200252. doi: 10.1098/rsob.200252, PMID: 33022193 PMC7653356

[ref61] Skelton J. Johnson A. J. Jusino M. A. Bateman C. C. Li Y. Hulcr J. (2019). A selective fungal transport organ (mycangium) maintains coarse phylogenetic congruence between fungus-farming ambrosia beetles and their symbionts. Proc. Royal Soc. B 286:20182127. doi: 10.1098/rspb.2018.2127, PMID: 30963860 PMC6367168

[ref62] Steininger M. S. Hulcr J. Šigut M. Lucky A. (2015). Simple and efficient trap for bark and ambrosia beetles (Coleoptera: Curculionidae) to facilitate invasive species monitoring and citizen involvement. J. Econ. Entomol. 108, 1115–1123. doi: 10.1093/jee/tov014, PMID: 26470236

[ref64] Tuncer C. Kushiyev R. Erper I. (2017). Determination of fungal flora on *Anisandrus dispar* Fabricius and *Xylosandrus germanus* Blandford (Coleoptera: Curculionidae: Scolytinae). Acta Hortic. 1226, 391–398. doi: 10.17660/ActaHortic.2018.1226.60

[ref65] van de Peppel L. J. J. Aanen D. K. Biedermann P. H. (2018). Low intraspecific genetic diversity indicates asexuality and vertical transmission in the fungal cultivars of ambrosia beetles. Fungal Ecol. 32, 57–64. doi: 10.1016/j.funeco.2017.11.010

[ref66] Vega F. E. Biedermann P. H. (2020). On interactions, associations, mycetangia, mutualists and symbiotes in insect-fungus symbioses. Fungal Ecol. 44:100909. doi: 10.1016/j.funeco.2019.100909

[ref68] Weisskopf L. Schulz S. Garbeva P. (2021). Microbial volatile organic compounds in intra-kingdom and inter-kingdom interactions. Nat. Rev. Microbiol. 19, 391–404. doi: 10.1038/s41579-020-00508-1, PMID: 33526910

[ref69] Wertheim B. van Baalen E. J. A. Dicke M. Vet L. E. (2005). Pheromone-mediated aggregation in nonsocial arthropods: an evolutionary ecological perspective. Annu. Rev. Entomol. 50, 321–346. doi: 10.1146/annurev.ento.49.061802.123329, PMID: 15355243

[ref70] White T. J. Bruns T. Lee S. Taylor J. (1990). “Amplification and direct sequencing of fungal ribosomal RNA genes for phylogenetics” in PCR protocols: A Guide to Methods and Applications. eds. Innis M. A. Gelfand D. H. Sninsky J. J. White T. J. (Academic Press), vol. 18, 315–322.

[ref71] Xia J. Psychogios N. Young N. Wishart D. S. (2009). Metabo analyst: a web server for metabolomic data analysis and interpretation. Nucleic Acids Res. 37, W652–W660. doi: 10.1093/nar/gkp356 19429898 PMC2703878

[ref72] Xu L. Lou Q. Cheng C. Lu M. Sun J. (2015). Gut-associated bacteria of *Dendroctonus valens* and their involvement in verbenone production. Microb. Ecol. 70, 1012–1023. doi: 10.1007/s00248-015-0625-4, PMID: 25985770

[ref73] Xu H. Turlings T. C. (2018). Plant volatiles as mate-finding cues for insects. Trends Plant Sci. 23, 100–111. doi: 10.1016/j.tplants.2017.11.004, PMID: 29229187

[ref74] Yang Q. Ye H. Zhang M. (2008). Composition and variety of the ambrosia fungi associated with ambrosia beetle, *Xylosandrus germanus* (Blandford) (Coleoptera: Scolytidae). Acta Entomol. Sin. 51, 595–600.

[ref75] Zhao T. Axelsson K. Krokene P. Borg-Karlson A. K. (2015). Fungal symbionts of the spruce bark beetle synthesize the beetle aggregation pheromone 2-methyl-3-buten-2-ol. J. Chem. Ecol. 41, 848–852. doi: 10.1007/s10886-015-0617-3, PMID: 26302987

