# Supplementary material for: Volatiles of fungal cultivars act as cues for host-selection in the fungus-farming ambrosia beetle Xylosandrus germanus
Source: Front Microbiol. 2023 Apr 14;14:1151078. doi: 10.3389/fmicb.2023.1151078 (PMC10140376; doi:10.3389/fmicb.2023.1151078)
Supplement: Supplementary file 3 [file Data_Sheet_1.docx]

Supplementary Material

Volatiles of fungal cultivars act as cues for host-selection in the fungus-farming ambrosia beetle *Xylosandrus germanus*

Antonio Gugliuzzo^1*^, Jürgen Kreuzwieser^2^, Christopher M. Ranger^3^, Giovanna Tropea Garzia^1^, Antonio Biondi^1^, Peter H. Biedermann^4*^

^1^Department of Agriculture, Food and Environment, University of Catania, Via Santa Sofia 100, 95123, Catania, Italy

^2^Ecosystem Physiology, University of Freiburg, Georges-Köhler-Allee 53/54, 79110 Freiburg, Germany

^3^USDA-Agricultural Research Service, Horticultural Insects Research Laboratory, 1680 Madison Ave., Wooster, OH 44691, USA

^4^Forest Entomology and Protection Research Group, University of Freiburg, D-79100 Freiburg, Germany

*** Correspondence:** Antonio Gugliuzzo; Peter H. Biedermann
antonio.gugliuzzo@phd.unict.it; peter.biedermann@forento.uni-freiburg.de

Keywords: *Ambrosiella grosmanniae*, fungal volatiles, mutualism, MVOCs, symbiosis, aggregation pheromone, Xyleborini

## Supplementary Figures

**Supplementary Figure S1.** Ethanol-baited trap used to collect and maintain living *Xylosandrus germanus* dispersing within a natural forest in Stegen-Wittental, Freiburg, Germany between April and June 2021. Traps were hung at ~80 cm from the ground and fixed to the trunk of beech trees. A vial containing 80% ethanol, previously arranged in the laboratory, was hung inside each trap and refilled at each sampling date.

**
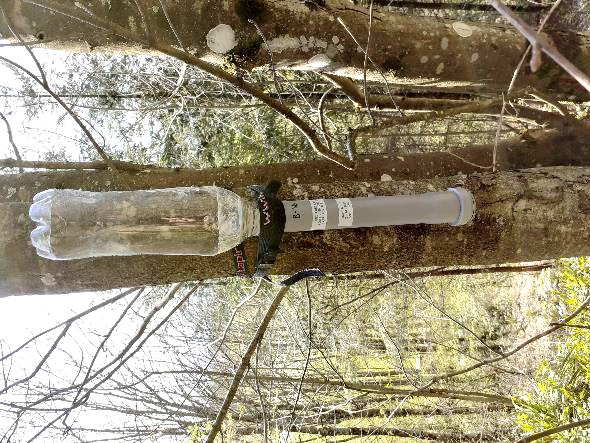
**

**Supplementary Figure S2.** Still-air bioassay used to assess the preference of *Xylosandrus germanus* for volatiles emitted by beetle-associated fungi.

**Supplementary Figure S3.** Bioassay arena used to test the effect of wood pre-infestation by *X. germanus* (1^st^ in figure) on the preference of a 2^nd^ *X. germanus* beetle released 6 days later. The arenas consisted of plastic boxes (114 × 114 × 58 mm) where *X. germanus* females were allowed to choose between a pre-infested beech bolt and a non-infested control bolt. Holes (diam. ~1 mm) were drilled at a distance of ~5 mm apart into two of the opposing walls and on the lid of each box to facilitate air exchange and gradients of volatiles. The same arena was used to test the boring preference behavior of *X.* *germanus* for a beech bolt pre-inoculated with the primary fungal mutualist *A. grosmanniae* *vs*. a non-inoculated bolt.

**Supplementary Figure S4.** a) Longitudinal section of a beech bolt revealing a *Xylosandrus germanus* brood chamber at 6 days after the foundress beetle initiated boring into the bolt. A beetle was first released in an arena (Fig. S3) containing two bolts and permitted to initiate boring into one of the bolts. During 6 days of incubation, the foundress was able to cultivate the primary fungal mutualist and oviposit eggs within the brood chamber. The boring preference of a 2^nd^ beetle for a bolt pre-infested by conspecifics vs. a non-infested control bolt was then tested. b) Beech bolt artificially infected by *Ambrosiella grosmanniae* and used to test the effect of wood pre-infection by the fungal mutualist on beetle boring preference. Six days after fungal inoculation, *A. grosmanniae* mycelia was confirmed to have grown around an artificially drilled hole in the bolt (as indicated by an arrow).

**Supplementary Figure S5.** Relative abundance of colony-forming units of different fungal morphotypes isolated from dispersing *Xylosandrus germanus*. Fungal isolation was conducted on beetle females caught by means of ethanol-baited traps at the Forest Entomology and Protection Institute, Stegen-Wittental, Freiburg (Germany) between April and June 2021. Colonies of isolated fungi (growing on YEMA (yeast extract malt agar) plates) showing different morphology were counted and the relative percentage proportions calculated.
